# Molecular characterization of polyphenol oxidase between small and large leaf tea cultivars

**DOI:** 10.1038/s41598-022-17184-1

**Published:** 2022-07-27

**Authors:** Chung‑Tse Chen, Chin-Ying Yang, Jason T. C. Tzen

**Affiliations:** 1 Graduate Institute of Biotechnology, National Chung Hsing University, Taichung, 40227 Taiwan; 2 Department of Agronomy, National Chung Hsing University, Taichung, 40227 Taiwan; 3 Smart Sustainable New Agriculture Research Center (SMARTer), National Chung Hsing University, Taichung, 40227 Taiwan

**Keywords:** Biotechnology, Molecular biology, Plant sciences

## Abstract

Tea is a widely consumed beverage prepared using the fresh leaves of *Camellia sinensis* L. Tea plants are classified into small- and large-leaf varieties. Polyphenol oxidase (PPO), a crucial enzyme in tea manufacturing, catalyzes essential phenolic metabolites into different derivatives. To compare the molecular characteristics of CsPPO between cultivars, we cloned the full-length sequence of *CsPPO* cDNA from four representative tea cultivars in Taiwan. Amino acid sequence alignment analyses indicated that CsPPO is highly conserved. PPO exhibited similar enzymatic activity in different tea cultivars. Quantitative real-time polymerase chain reaction revealed no significant differences in the *CsPPO* transcript level between the small- and large-leaf varieties. However, tea harvested in summer and from low-altitude areas had a higher *CsPPO* transcript level than that harvested in winter and from high-altitude areas. Regulation of *CsPPO* by temperature was more significant in the small-leaf variety than in the large-leaf variety. The content of flavonoids and the expression of chalcone synthase, anthocyanidin synthase, and anthocyanidin reductase genes involved in the tea flavonoid biosynthesis pathway were higher in the large-leaf than in the small-leaf varieties, suggesting that the large-leaf variety had a higher antioxidative capacity than did the small-leaf variety. Our study compared the molecular properties of *CsPPO* between two tea varieties and clarified the physiological role of PPO in tea.

## Introduction

Tea is the most widely consumed beverage worldwide. Tea plants (*Camellia sinensis*) contain numerous secondary metabolites, including polyphenols, flavonoids, alkaloids, amino acids, terpenoids, and vitamins. Polyphenols are the major secondary metabolites present in tea and account for approximately 36% of the dry weight of tea leaves^1^. Catechins comprise >90% of phenolic compounds in tea^2^, including epicatechin, epicatechin gallate, epigallocatechin, epigallocatechin gallate, and gallocatechin^3,4^. Moreover, catechin exhibits antioxidative activity, improves tea quality, and provides various health benefits^5^. Quercetin, kaempferol, myricetin, and their glycoside derivatives are the major flavonols present in tea^3^. Moreover, the flavonoid theaflavin is produced through the fermentation of catechin in tea^6^ and is closely related to black tea quality. Caffeine and theophylline are common alkaloids present in tea that stimulate the central nervous system. Furthermore, tea contains numerous amino acids that impart tea with an umami taste^7^.

Polyphenol oxidase (PPO) is a copper-containing enzyme present in many species. PPO oxidizes the hydroxyl group of phenolic compounds to produce quinones. PPO is related to the biotic and abiotic stress resistance of plants^8–10^. Moreover, PPO causes the browning of fruits and vegetables. In addition, PPO catalyzes the oxidation of endogenous polyphenols in fruits and vegetables to produce melanin, which adversely affects the nutrition, flavor, and appearance of plant products^11^. However, the role of PPO is crucial in certain plants, such as tea. The tea plant is rich in tea polyphenols. Catechins are the most vital bioactive compound in tea and increase the antioxidant capacity in humans. The coprecipitation of catechins and proteins is responsible for the bitterness and astringency of tea. PPO oxidizes catechins to produce theaflavins and thearubigins^12^, which greatly reduce the astringent taste and contributing to the color of black tea. Thus, PPO substantially affects the taste, color, and flavor of tea.

*C. sinensis* var. *sinensis* and *C. sinensis* var. *assamica* are the two most commonly used varieties for preparing tea. Many differences are noted between the characteristics of these two varieties, with leaf size being among the most prominent differences. The leaf of *C. sinensis* var. *sinensis* (a small-leaf variety) is smaller and thicker than that of *C. sinensis* var. *assamica* (a large-leaf variety). The average leaf length and area are 10.4 cm and 48.2 cm^2^, respectively, for *C. sinensis* var. *sinensis* and 13.5 cm and 74.5 cm^2^, respectively, for *C. sinensis* var. *assamica*^13^. In addition to leaf size, the leaf structure differs between the two varieties. The small-leaf variety has thick multilayered palisade mesophyll cells in leaves, whereas the large-leaf variety has single-layered palisade mesophyll cells in leaves. Moreover, spongy mesophyll cells are thicker and loosely arranged in the large-leaf variety than in the small-leaf variety. These differences may have resulted from evolutionary adaptation^14,15^. Multilayered palisade mesophyll cells enhance the endurance level of plants when exposed to low temperatures and intensive sunlight^16^ and enable the small-leaf variety to grow at high altitudes^17,18^. However, the large-leaf variety that possesses loosely arranged thick spongy mesophyll cells exhibits high evapotranspiration, which is crucial for growth in high-temperature regions, such as low-altitude and tropical regions. Moreover, the chemical composition differs between the two varieties. The large-leaf variety contains more solids compared with the small-leaf variety, including tea polyphenols and caffeine, which contribute to the astringency and bitterness of tea infusions^19,20^. The small-leaf variety contains higher contents of carotenoid and lutein than does the large-leaf variety, and these terpene compounds further break down into small volatile compounds, such as ionone, theaspirone, and dihydroactinidiolide^21^. The difference on leaf structures and metabolites composition between various tea variety not only decides the physiological properties of the tea plant but also affects the tea making method and the taste of tea infusion.

Taiwan is located in subtropical and tropical regions in Southeast Asia. Because Taiwan is a mountainous island country, its diverse terrain and warm weather are suitable for tea cultivation. Environmental factors affect the polyphenol content and overall chemical composition of tea. The total catechin content in tea is higher in autumn than in spring and gradually decreases with increasing altitude^22,23^. Taiwan’s diverse terrain and warm climate result in the growth of tea plants with unique characteristics. The tea cultivars Chin-shin oolong (Chin-shin) and Taiwan tea experiment station (TTES) No. 12 belong to the small-leaf variety. Chin-shin is currently the largest planting area tea cultivar in Taiwan. The ability of Chin-shin to grow at low temperatures makes it suitable for cultivation in the mountainous areas of Taiwan. The small-leaf cultivar is predominantly used for preparing oolong tea. The cultivars TTES No. 8 and TTES No. 18 belong to the large-leaf variety and are mainly grown in the flat hilly regions of central Taiwan; these cultivars are predominantly used for preparing black tea.

The tea industry in Taiwan has been developing for more than 200 years. Teas with unique flavors produced in Taiwan occupy a niche in the global tea market. Different variety of tea have genetic backgrounds that affect the appearance and physiological traits of the leaves as well as the constitution of metabolites and the activities of enzymes. These genetic differences have a direct and substantial effect on the production of tea in addition to affecting the appearance and physiological traits of the leaves. The majority of current research on tea focuses mostly on the metabolite analyses, and the molecular information of critical enzyme, PPO, between different genetic background of tea remain scarce. Because of the high natural landscape and genetic diversity among tea cultivars grown in Taiwan, we are able to explore the molecular characteristics and elucidate mechanisms through which crucial enzyme genes are regulated in tea. In this study, we examined differences in molecular characteristics, such as enzyme activity, and the transcriptional regulation of PPO between tea cultivars (two small-leaf and two large-leaf variety). We cloned *CsPPO* cDNA sequences from four varieties and compared their amino acid sequences. Moreover, we examined the effects of season, altitude, and temperature on the expression level of *CsPPO* in tea plant. We determined the contents of total phenol, flavonoids, and anthocyanins in the four varieties. Our results provide evidence of the differential regulation of *CsPPO *in the small-leaf and large-leaf varieties.

## Results

### CsPPO sequences are highly conserved in small- and large-leaf tea cultivars

**Figure 1:**
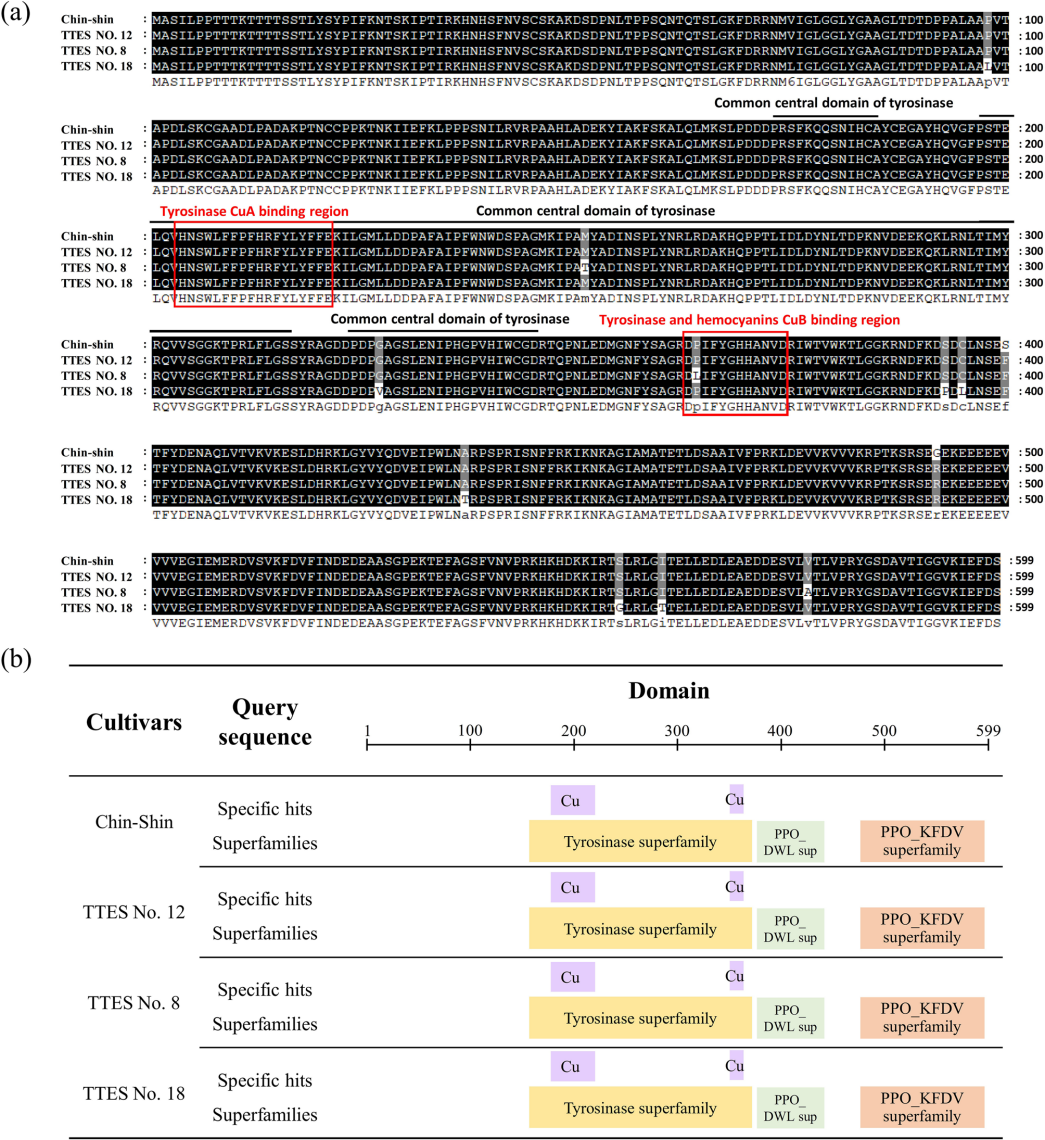
Sequence alignment and functional domain prediction of CsPPO sequences in different cultivars. (**a**) Alignment of amino acid sequences of CsPPO in small- and large-leaf varieties. Black and gray shades indicate sequence conservations of 100% and > 80%, respectively. Sequences were aligned using the ClustalW algorithm. Black lines above the sequences indicate the common central domain of tyrosinase. Red boxes indicate the tyrosinase CuA-binding region HNSWLFFPFHRFYLYFFE and the tyrosinase and hemocyanin CuB-binding region DPIFYGHHANVD. (**b**) Predicted functional domains on the CsPPO sequences. Squares in different color indicate the position of conserved domain of protein families on the sequences. Purple squares represented two cooper ion binding regions of tyrosinase.

The *CsPPO* sequences of the cultivars were determined through gene cloning and sequencing. The open reading frame of *CsPPO* cDNA was 1800 bp and encoded a polypeptide of 599 amino acids. The mRNA sequences of *CsPPO* in the tea cultivars were registered in the National Center for Biotechnology Information (NCBI) database with the GeneBank accession numbers MZ442717: https://www.ncbi.nlm.nih.gov/nuccore/MZ442717; MZ442718: https://www.ncbi.nlm.nih.gov/nuccore/MZ442718; MZ442719: https://www.ncbi.nlm.nih.gov/nuccore/MZ442719; MZ442720: https://www.ncbi.nlm.nih.gov/nuccore/MZ442720 for Chin-shin, TTES No. 12, TTES No. 8, and TTES No. 18, respectively. The sequences were aligned using ClustalW (https://www.genome.jp/tools-bin/clustalw; Fig. 1a). The amino acid sequences of CsPPO were highly conserved and exhibited up to 98% similarity among the four cultivars. The online Motif Scan tool (https://myhits.sib.swiss/cgi-bin/motif_scan) was used to predict functional domains on the amino acid sequences of CsPPO; the results indicated that all the CsPPO sequences contained the common central domain of tyrosinase and two conserved copper-binding sites (Fig. 1b). These results imply that the four tea cultivars may have a similar protein structure and activity.

### Transcript levels of *CsPPO* in leaf tissues differed among cultivars

**Figure 2:**
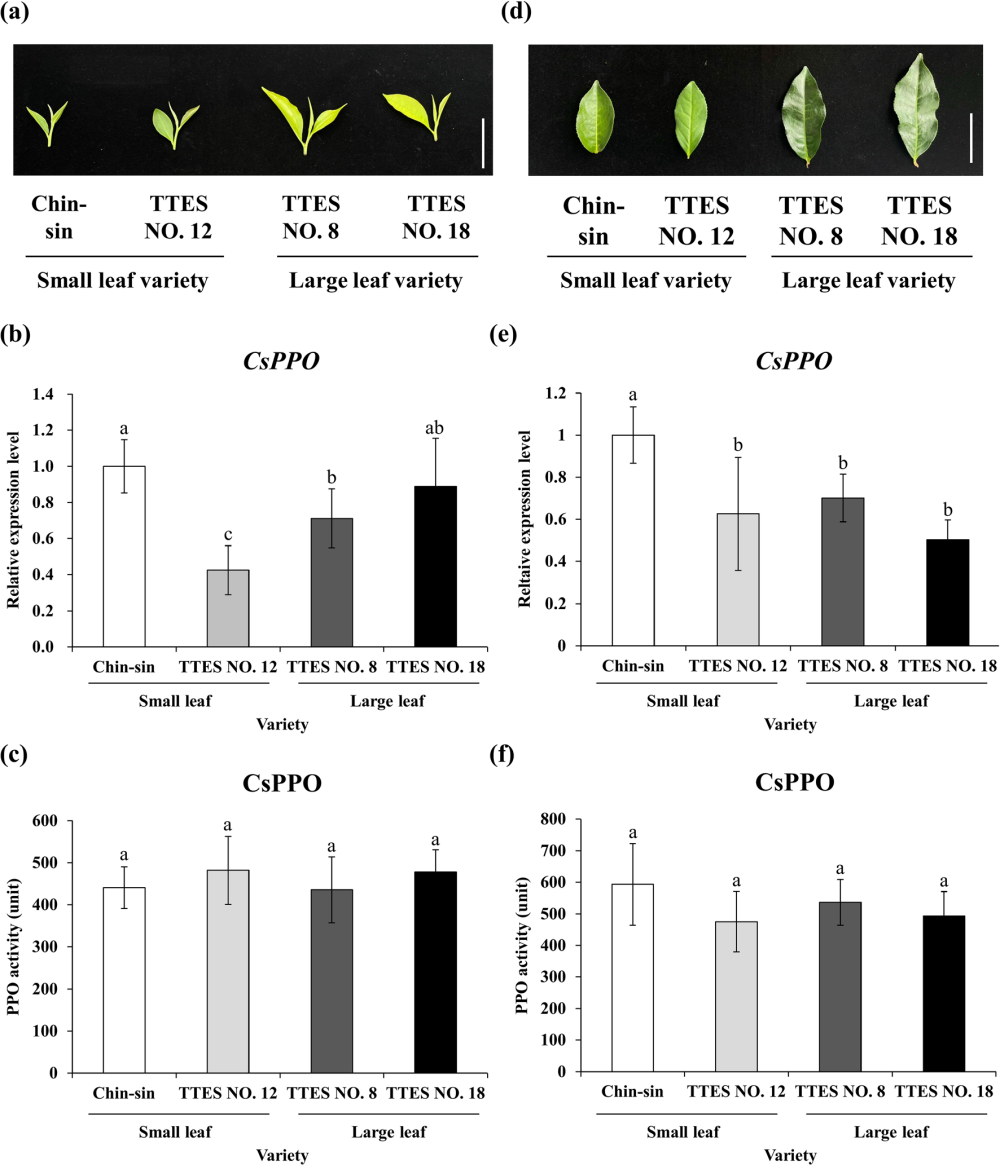
Leaf appearance, *CsPPO* transcript level, and CsPPO enzyme activity in the young and old leaves of small- and large-leaf varieties. Photo of (**a**) young and (**b**) old leaves of small- and large-leaf varieties. *CsPPO* transcript levels of the (**b**) young and (**e**) old leaf tissues of small- and large-leaf varieties. PPO enzyme activity in (**c**) young and (**f**) old leaf tissues of small- and large-leaf varieties. Total RNA was extracted from the young and old leaves of each variety. *CsPPO* transcript levels were measured using qRT-PCR and normalized to that of 18S rRNA. One unit of PPO activity was defined as the amount of enzyme required to metabolize pyrocatechol into a micromole of benzoquinone per minute. Values are presented as the mean ± SD of three biologically independent experiments. Different letters indicate significant difference between varieties assessed using the LSD post hoc test (*p* < 0.05). Bar = 5 cm.

PPO oxidizes polyphenol in tea leaves and thus substantially affects the chemical composition and color of tea infusions as well as tea quality. To compare the molecular characteristics of PPO among the tea cultivars, we examined the transcription level and enzymatic activity of CsPPO in the leaf tissue of the four cultivars. Figure 2a,d present differences between the young and old leaves of the small- and large-leaf varieties. We performed quantitative real-time polymerase chain reaction (qRT-PCR) to examine the *CsPPO* transcript level in the tea cultivars. Chin-shin had the highest *CsPPO* transcript level in both young and old leaf tissues. TTES No. 12 had the lowest *CsPPO* transcript level in the young leaf tissue. The *CsPPO* transcript levels were identical in the old leaf tissues of TTES No. 12, TTES No. 8, and TTES No. 18. The results indicated that although *CsPPO* transcript levels varied among the cultivars, the levels did not markedly differ between the small- and large-leaf cultivars (Fig. 2b,e).

To compare PPO enzymatic activity among the cultivars, we performed a PPO enzyme activity assay. PPO extracted from the four cultivars exhibited the same enzymatic activity in both young and old leaf samples, indicating that the oxidation capacity of PPO was identical in the young and old leaves of these cultivars (Fig. 2c,f).

### Small-leaf variety exhibited different expression patterns of *CsPPO* in samples depending on season and altitude

**Figure 3:**
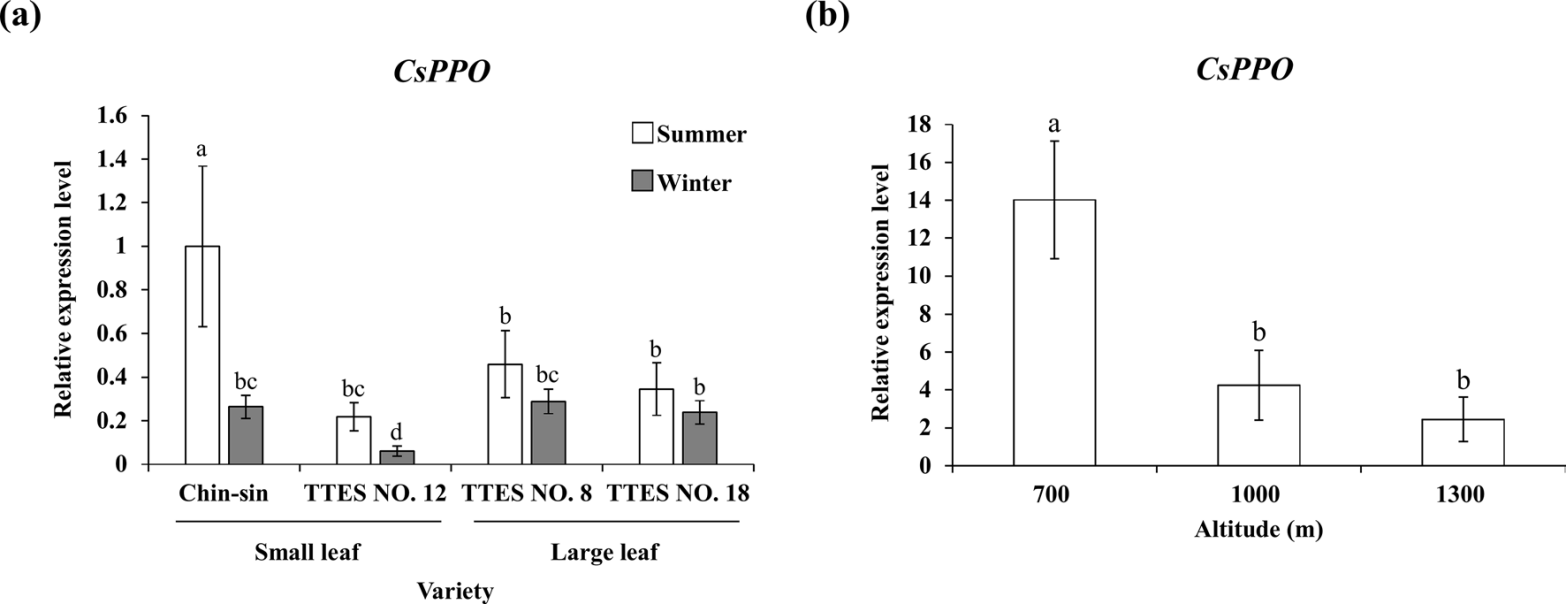
*CsPPO* transcript level in different harvesting seasons and plantation altitudes. Samples of each variety were collected in the summer and winter of 2018. The Chin-shin samples from different plantation altitudes were collected from tea gardens in Alishan, Chiayi. Total RNA was extracted from the young leaves of each sample. *CsPPO* transcript levels were measured using qRT-PCR and normalized to that of 18S rRNA. Values are presented as the mean ± SD of three biologically independent experiments. Different letters indicate significant difference between samples assessed using the LSD post hoc test (*p* < 0.05).

We compared the *CsPPO* transcript level in the young leaves of the tea cultivars grown in different seasons. Moreover, we examined the *CsPPO* transcript level in the young leaves of Chin-shin cultivated at different altitudes. The *CsPPO* transcript level in the young leaves of Chin-shin and TTES No. 12 were significantly higher in summer than in winter (Fig. 3a). However, the same expression pattern was not observed in TTES No. 8 and TTES No. 18. This result indicates that the *CsPPO* transcript level in the small-leaf variety was more sensitive to seasonal changes than that in the large-leaf variety.

Chin-shin is the major cultivar planted at high altitudes (above 1000 m) in Taiwan. We collected the young leaves of Chin-shin cultivated at different altitudes (700, 1000, and 1300 m) in Nantou, Taiwan. The total RNA was isolated from the samples, and qRT-PCR was performed to determine the *CsPPO* transcript level. Tea leaves grown at an altitude of 700 m had the highest *CsPPO* transcript level (Fig. 3b). The *CsPPO* transcript level was negatively correlated with altitude. The *CsPPO* transcript level tended to be upregulated in the cultivars grown at high temperatures.

### Temperature regulates *CsPPO* expression in the bud of tea seedlings

**Figure 4:**
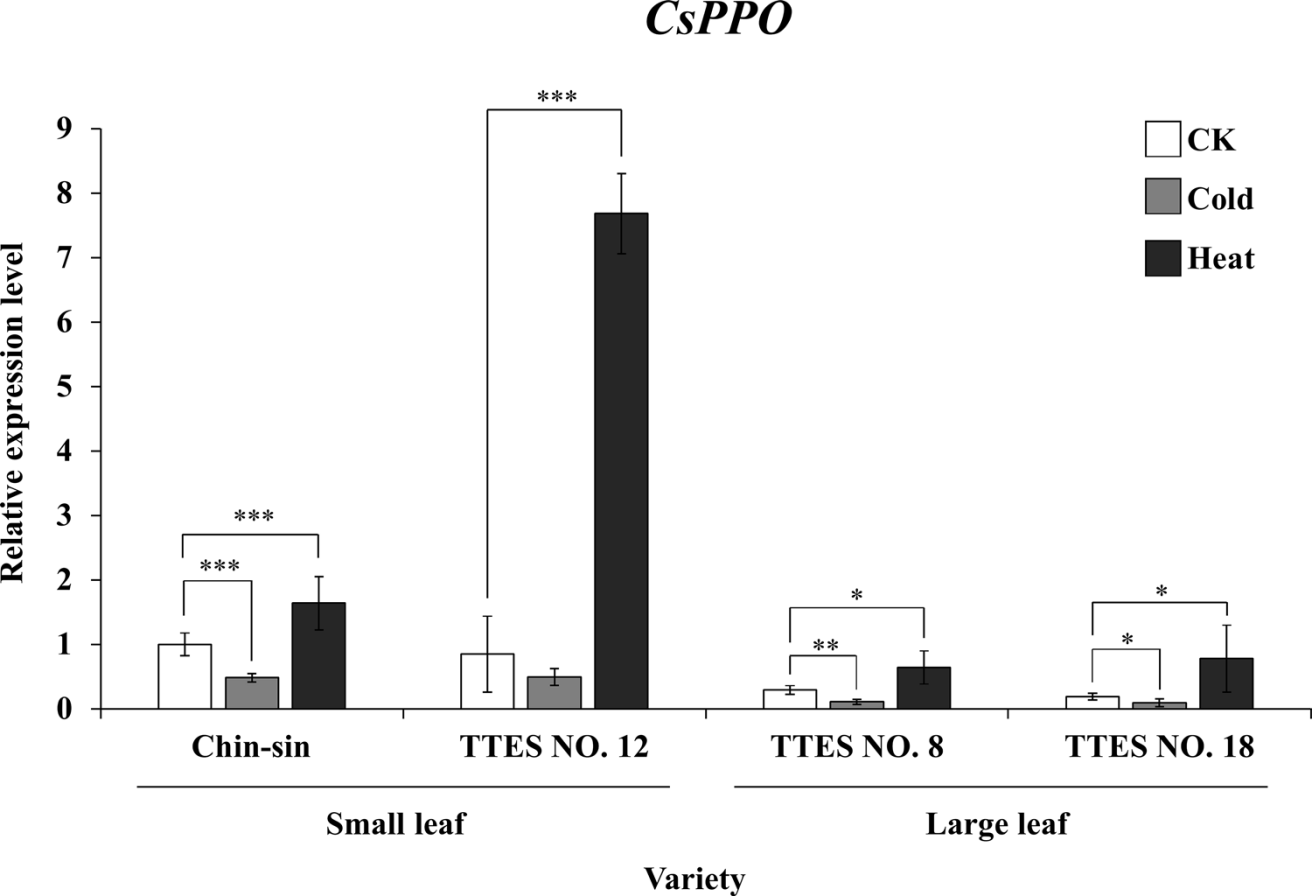
*CsPPO* regulation by different abiotic stress in small- and large-leaf varieties. One-year-old seedlings of different varieties were subjected to CK (22 °C), cold (4 °C), and heat (35 °C) stress treatments. Total RNA was extracted from the young leaves of each sample. *CsPPO* transcript levels were measured using qRT-PCR and normalized to that of 18S rRNA. Values are presented as the mean ± SD of four biologically independent experiments (N = 4, n = 4). Asterisks indicate significant differences between treatments assessed using the LSD post-hoc test (*p < 0.05, **p < 0.01, ***p < 0.001).

*CsPPO* expression significantly differed between the seasons. To analyze the effects of environmental factors, particularly temperature, on the expression pattern of CsPPO in tea, we exposed tea seedlings to high temperature (35℃) and low temperature (4℃) for 1 day each. The results indicated that high temperature significantly upregulated the *CsPPO* transcript level in the large-leaf cultivars (*p* < 0.001) and small-leaf cultivars (*p* < 0.05). Low temperature inhibited *CsPPO* expression in all the cultivars except TTES No. 12 (Fig. 4).

### Total phenol, flavonoid, and anthocyanin contents and the transcript level of related genes in four tea cultivars

Polyphenols are the most abundant secondary metabolites in tea leaves. Phenolic compounds play a crucial role in plants. Furthermore, flavonoids and anthocyanins are phenolic compounds. Flavonoids exhibit antioxidant activity that prevents DNA or cell membrane damage due to environmental stress, such as that caused by UV exposure^24^. Moreover, phenolic compounds play an indispensable role in tea quality. PPO catalyzes the oxidation of catechins into theaflavins in tea leaves during tea manufacturing. The color and taste of tea significantly change after fermentation. We measured the total phenol, flavonoid, and anthocyanin contents of the young and old leaves of the four cultivars to compare PPO substrates among the cultivars.

**Figure 5:**
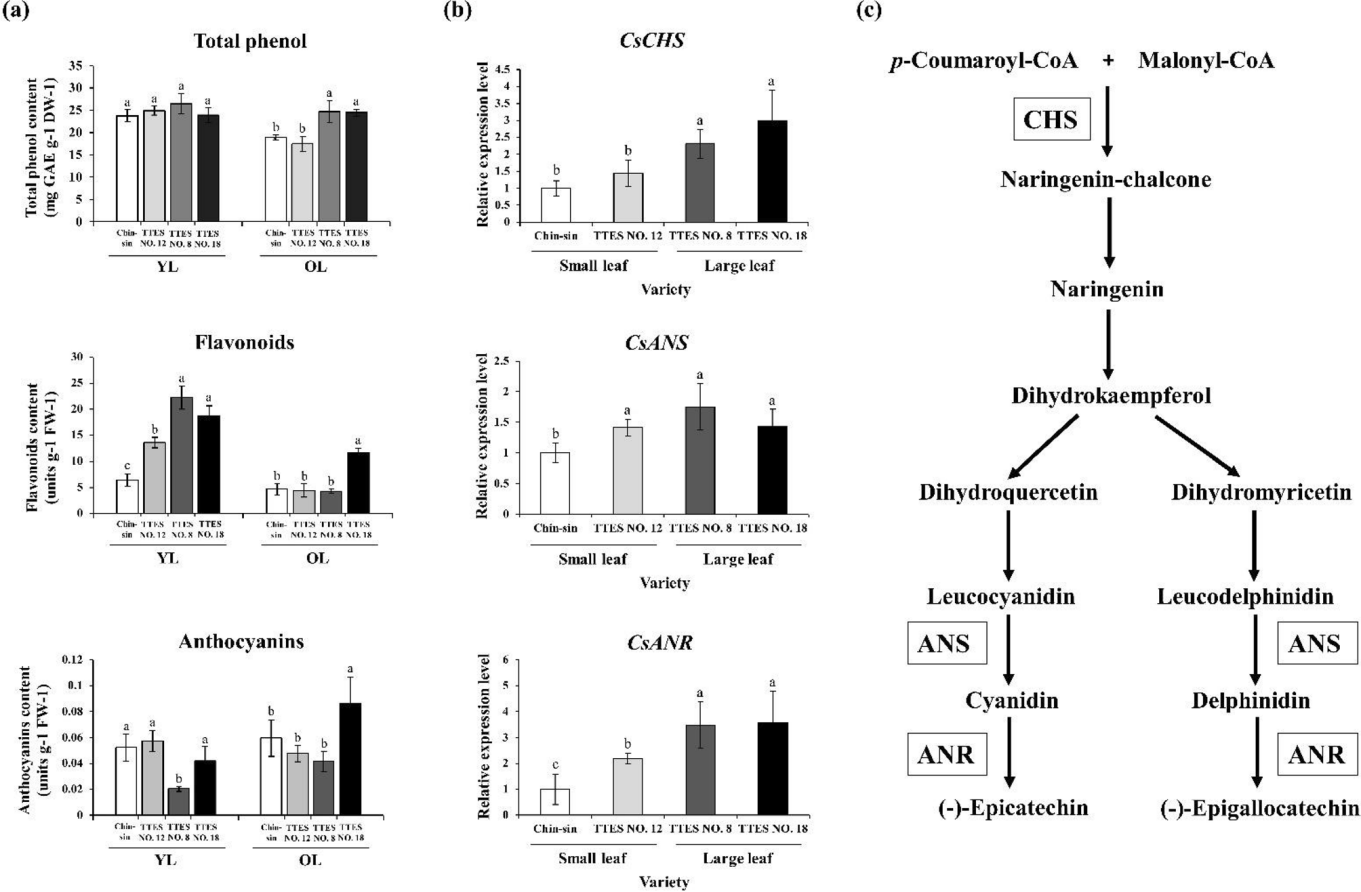
Comparison of secondary metabolite contents and transcript levels of flavonoid biosynthesis–related genes in small- and large-leaf varieties. (**a**) Contents of total phenol, flavonoids, and anthocyanins in the young and old leaf tissues of different tea varieties. (**b**) Transcript levels of *CsCHS*, *CsANS*, and *CsANR* in the young leaf tissues of different tea varieties. Transcripts levels were measured using qRT-PCR and normalized to that of 18S rRNA. (**c**) The flavonoid biosynthesis pathway in tea. Values are presented as the mean ± SD of three biologically independent experiments. Different letters indicate significant differences between varieties assessed using the LSD post-hoc test (*p* < 0.05). *ANS* anthocyanidin synthase; *ANR* anthocyanidin reductase; *CHS* chalcone synthase; *DW* dry weight; *FW* fresh weight; *GAE* gallic acid equilibrium.

The total phenol content in young leaf tissues did not significantly differ between the small- and large-leaf tea cultivars. However, the old leaf tissues of the large leaf cultivars (TTES No. 8 and TTES No. 18) had a higher total phenol content than did those of the small-leaf cultivars (Chin-shin and TTES No. 12; Fig. 5a). Furthermore, the young leaves of the large-leaf cultivars had a higher flavonoid content than did those of the small-leaf cultivars. With regard to the young leaves of these two small-leaf cultivars, TTES No. 12 had a higher flavonoid content than did Chin-shin. With regard to the old leaves of the cultivars, TTES No. 18 had a significantly higher flavonoid content than did the other cultivars (Fig. 5a). The young leaves of Chin-shin, TTES No. 12, and TTES No. 18 had a higher anthocyanin content than did those of TTES No. 8. However, the old leaves of TTES No. 18 had the highest anthocyanin content among the four cultivars, and the anthocyanin content did not significantly differ among the other three cultivars.

We investigated the transcript levels of genes involved in the flavonoid synthase pathway, namely *chalcone synthase* (*CsCHS*), *anthocyanidin synthase* (*CsANS*), and *anthocyanidin reductase* (*CsANR*; Fig. 5c). Total RNA was extracted from the young leaves of the four cultivars. To analyze the relative transcript levels of the genes, qRT-PCR was performed. The large-leaf cultivars had higher transcript levels of *CsCHS* and *CsANR* than did the small-leaf cultivars. Furthermore, *CsANS* expression appeared to be higher in the large-leaf cultivars than the small-leaf cultivars (Fig. 5b).

## Discussion

The taste of tea largely depends on the manufacturing process. Fermentation essentially determines the chemical composition of tea. PPO is a major enzyme involved in tea fermentation^4,6^, indicating the importance of PPO in tea production. To investigate the molecular characteristics of CsPPO in different tea cultivars, we cloned sequences from four Taiwanese tea cultivars. PPO is an enzyme widely present in nature. The sequence homology of PPO is directly related to the closeness among species^25^. Furthermore, the amino acid sequences of CsPPO in our study exhibited a high degree of similarity among the four tea cultivars (Fig. 1). Although the sequence length varied among plants, the average nucleotide sequence of the PPO gene in different plants was approximately 1800 bp, and the translated PPO precursor protein was approximately 600 amino acid long^26^, which is consistent with CsPPO sequences obtained in this study.

In Taiwan, the tea grown at different altitudes depends on cultivar. The temperature is higher in low-altitude areas, resulting in severe problems related to pests and diseases. PPO is considered to be primarily related to the resistance of plants to pests and disease. The absorption of quinones produced by PPO and reactive oxygen species produced through the redox cycle of quinones exert bactericidal and fungicidal effects on pests^27^. Quinones produced through the PPO reaction can catalyze the further modification of proteins with amine, phenol, and sulfur groups and reduce the nutritional value of the plant for pests. Our study revealed that *CsPPO* expression in tea is induced by high temperature and inhibited by low temperature (Figs. 3 and 4). These results support the aforementioned hypothesis. The differences in the *CsPPO* transcript level between the small- and large-leaf varieties under the same high-temperature condition (35℃) might be because the small-leaf variety has adapted to the high-altitude area with low temperature, making high temperature a stress condition for the small-leaf variety. By contrast, for the large-leaf variety, which is typically planted in low-altitude areas with warm temperatures, 35℃ is not a severe stress condition.

Phenolic compounds are the key metabolites in tea. The content of polyphenols in tea directly affects the suitability of cultivars for tea manufacturing. The tea leaves of the small-leaf variety contain fewer phenolic compounds, and the tea grown in areas with low temperatures is used to prepare lightly fermented oolong tea. In Taiwan, the small-leaf cultivar Chin-shin is mostly used to make oolong tea with high fragrance. The quality and price of oolong tea prepared using Chin-shin cultivated in winter are higher than those of oolong tea prepared using Chin-shin cultivated in summer. The leaves of the large-leaf variety, which contain a high polyphenol content, are used to prepare fully fermented black tea. The maximum fermentation catalyzed by PPO not only enables the conversion of a high amount of polyphenols to theaflavin but also eliminates the astringent taste imparted by catechins.

Phenolic compounds in plants play a crucial role in growth control and exhibit antioxidative, structural, attractant, signaling, and protective functions^28^. The chemical composition of tea leaves is affected by numerous environmental factors, such as sunlight, rainfall, and temperature. Tea leaves differ in their composition of phenolic compounds depending on the season and the altitude of cultivation. Catechin gallate levels are higher in leaves harvested in warmer seasons^29^, and the catechin content in tea is inversely correlated with the cultivation altitude^22^. This finding might be related to the physiological functions of tea polyphenols, such as their antioxidative effect and defense against diseases or pests. In old leaf tissues, the polyphenol content was significantly higher in the large-leaf variety than in the small-leaf variety (Fig. 5a). Furthermore, the transcript levels of genes involved in the tea flavonoid synthesis pathway indicated that polyphenols are synthesized more vigorously in the large-leaf variety than the small-leaf variety (Fig. 5b). These results demonstrate that the large-leaf variety might be more suitable than the small-leaf variety for planting at a low-altitude area with high temperature.

## Conclusion

We compared the molecular characteristics of CsPPO and the polyphenol content among the four representative tea cultivars grown in Taiwan. These results provide evidence regarding the adaptation of the small- and large-leaf tea varieties to different environments. Studies on PPO in plants have mostly focused on defense against diseases and pests, and few studies have discussed the role of PPO during abiotic stress. Our study examined *CsPPO* regulation in tea seedlings during different temperature stresses, providing the basis for further studies on the physiological role of PPO in plants.

## Methods

### Plant material

**Figure 6:**
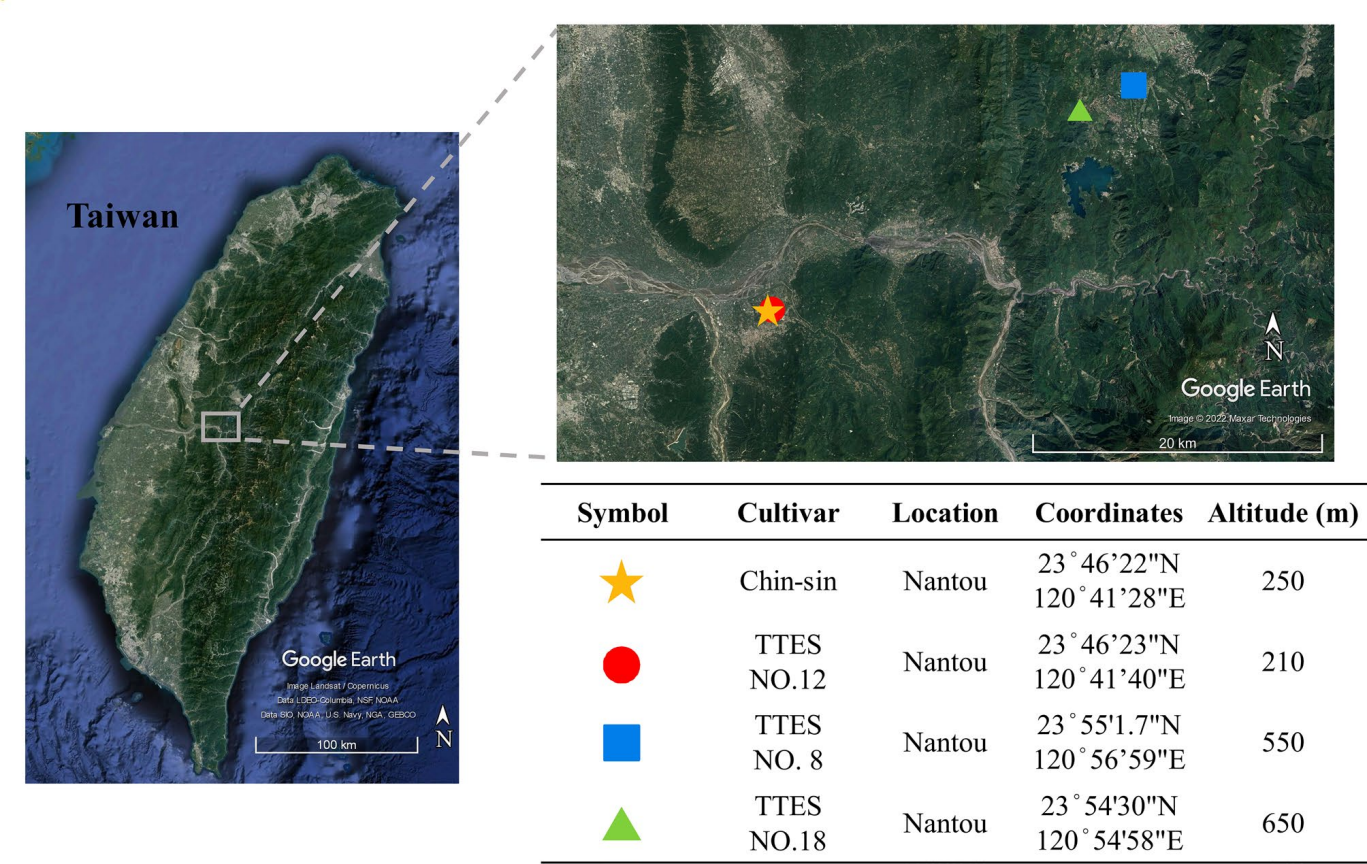
Geographic location and information of tea gardens of different cultivars. Tea samples were collected from these locations in Nantou, Taiwan. Different symbols represent the tea gardens of different cultivars. Base imagery was captured from Google Earth. Map data source: Google, LDEO-Columbia, NSF, NOAA, SIO, U.S. NAVY, NGA, GEBCO, Maxar Technologies.

The tea [*C. sinensis* (L.) Kuntze] cultivars Chin-shin, TTES No. 12, TTES No. 8, and TTES No. 18 were used in this study. The cultivars were planted in different tea gardens. Tea plant samples were collected from a tea farm. Nantou is the predominant tea cultivation area in Taiwan. To prevent interference from environmental factors, such as weather and soil composition, tea samples were collected from adjacent tea gardens in Nantou, Taiwan (Fig. 6). The map was captured from the Google Earth online service Map data: Google, Maxar Technologies. The links below are the views we used in Fig. 6https://earth.google.com/web/search/%e5%8d%97%e6%8a%95%e7%b8%a3%e9%ad%9a%e6%b1%a0%e9%84%89%e6%97%a5%e6%9c%88%e6%bd%ad/@23.5386579,121.01366748,2559.28282025a,6RbZOaMl5A and https://earth.google.com/web/search/%e5%8d%97%e6%8a%95%e7%b8%a3%e9%ad%9a%e6%b1%a0%e9%84%89%e6%97%a5%e6%9c%88%e6%bd%ad/@23.82078454,120.8182327,273.46785895a,54753.98344355d,0h,22.39691261t,0r/data=CigiJgokCUb4VwmLxDdAEaI9iTP9ozdAGVHS2xCDRV5AIR-RbZOaMl5A. All selected gardens had similar weather conditions, including temperature (mean annual temperature: 22 °C) and rainfall (annual precipitation: 2600 mm). In 2017, young leaf (one-tip two leaf) and old leaf (a fully developed leaf on a lignified branch) samples were frozen at − 196 °C in liquid nitrogen immediately after being plucked and stored at − 80 °C until further experimental use. Our study complied with relevant institutional, national, and international guidelines and legislation.

### Cloning and sequencing of CsPPO from different cultivars

The mRNA sequences of *CsPPO* in various plants were obtained from NCBI (https://www.ncbi.nlm.nih.gov/). Because of a high percent identity of CsPPO sequences among different tea cultivars (Fig. S1), we designed specific primers for the conserved ORF of *CsPPO* sequences. PCR involved the following steps: initial denaturation at 94 °C for 10 min, followed by 35 cycles at 94 °C for 30 s, 45 °C for 30 s, and 72 °C for 1 min, with a final extension at 72 °C for 7 min. PCR products were subsequently subjected to electrophoresis on 1% Tris-Borate-EDTA agarose gel to confirm product recovery. Subsequently, we used the PCR clean-up and gel extraction kit (GeneDireX, USA) for product extraction and purification. The purified product was linked with the pGEM-T Easy Vector, and the resulting ligation mixture was transformed into *Escherichia coli* cells to amplify the product sequence. The transformed *E. coli* cells were grown on Luria–Bertani medium containing ampicillin (30 mg/mL). The plasmids of the selected *E. coli* were purified using the plasmid miniprep purification kit (GeneMark, Taiwan) and then sequenced to obtain the *CsPPO* sequences of the cultivars (Table S1).

### qRT-PCR

Total RNA was extracted from the leaf samples by using the plant total RNA purification kit (GeneMark, Taiwan). The quality and concentration of the extracted RNA were analyzed using a NanoDrop Lite spectrophotometer (Thermo Fisher Scientific, USA). The extracted total RNA was then reverse transcribed using the M-MLV first-strand synthesis kit (Invitrogen, USA) to obtain cDNA. The sequences and primers of the genes of interest were obtained from the NCBI database. We performed qRT-PCR on the Bio-Rad instrument (CFX Connect, USA) by using the KAPA SYBR FAST qPCR master mix (KapaBiosystems, USA). The 10-μL PCR reaction volume consisted of 3 μL of cDNA (equivalent to 600 ng), 5 μL of 2× power SYBR mix, 0.4 μL of each primer to a final concentration of 400 nM, and 1.2 μL of ddH_2_O. The 18S rRNA gene was used as the housekeeping gene. Transcript levels were calculated using the 2^−ΔΔct^ method in Bio-Rad. At least three independent replicates were tested, and average values were obtained. Table S1 lists primer sequences used in this experiment.

### PPO activity assay

PPO enzymatic activity was examined using catechol as the exogenous substrate based on the method reported by Tram (2015)^30^. The tea sample (0.1 g) was ground using a mortar and pestle and mixed with 1 mL of sodium phosphate buffer (pH 7.0). The mixture was stirred for 30 min at 4 °C and centrifuged at 12,000 × *g* for 30 min at 4 °C. The supernatant was carefully collected and used as the crude extract. Enzyme-linked immunosorbent assay was performed in a 96-well plate by using 25 μL of the enzyme extract, 25 μL of 50 mM catechol in 50 mM phosphate buffer (pH 7.0), and 150 μL of 50 mM phosphate buffer. The absorbance of the enzymatic product benzoquinone at 420 nm was measured every minute by using a spectrophotometer (Beckman DU 650, USA). The blank control sample contained 1 mL of substrate solution. One unit of PPO activity was defined as the micromole of benzoquinone produced per minute.

### Temperature treatment

One-year-old seedlings of the cultivars Chin-shin, TTES No. 12, TTES No. 8, and TTES No. 18 were purchased from Nantou in Central Taiwan. The cultivars were grown at a high temperature (35 °C) and low temperature (4 °C) in growth chambers for 1 day, and the control group was maintained at 24 °C in a 16/8-h light/dark cycle. The buds of the seedlings were plucked and frozen in liquid nitrogen for further analyses (N = 4, n = 4).

### Total phenolic content

The total phenolic content was determined using the Folin–Ciocalteu method^31^. The tea leaves (0.1 g) were frozen in liquid nitrogen and ground into powder by using a mortar and pestle. The sample powder was mixed with 1 mL of EtOH (75%) and vortexed at room temperature for 2 h for extraction. After centrifugation at 10,000 × *g* for 10 min, the supernatant was collected and used as the ethanol extract. In a dark bottle, 4.2 mL of deionized water, 50 μL of the ethanol extract, and 250 μL of Folin–Ciocalteu’s phenol reagent (Merk, Germany) were allowed to react for 5 min. Subsequently, 500 μL of 20% Na_2_CO_3_ solution was added to stop the reaction. The absorbance of the final solution was determined at 765 nm by using a spectrophotometer (Metertec SP8001), and 50 μL of EtOH (75%) was used as a blank. The total phenolic content is expressed as milligrams of gallic acid equivalent per gram of dried waste.

### Flavonoid and anthocyanin contents

The flavonoid and anthocyanin contents of the leaves were measured using the method described by Kubo (1999)^32^ with slight modification. The tea leaves (0.1 g) were frozen in liquid nitrogen and ground into powder by using a mortar and pestle. Subsequently, 2 mL of 100 mM potassium phosphate buffer (pH 7.8) was mixed with the sample powder and vortexed. The mixture was then centrifuged at 16,000 × *g* at 4 °C for 30 min. The absorbance of the supernatant was measured at 320 and 600 nm by using a spectrophotometer (Metertec SP8001). A single absorbance unit was defined as the quantities of anthocyanins and flavonoids to exhibit an absorbance of 1 at 600 and 320 nm, respectively.

### Statistical analysis

A least significant difference (LSD) post-hoc test was conducted after analysis of variance for multiple comparisons between the samples. A *p* value of <0.05 indicated significant differences. All statistical analyses were performed using SAS version 9.4.

## Supplementary Information

Supplementary Information.

## Data Availability

The datasets generated and analyzed during the current study are available in the GenBank repository with the Accession Numbers MZ442717-MZ442720.
