# Publisher Correction: Molecular characterization of polyphenol oxidase between small and large leaf tea cultivars

**DOI:** 10.1038/s41598-022-18490-4

**Published:** 2022-08-18

**Authors:** Chung‑Tse Chen, Chin-Ying Yang, Jason T. C. Tzen

**Affiliations:** 1 Graduate Institute of Biotechnology, National Chung Hsing University, Taichung, 40227 Taiwan; 2 Department of Agronomy, National Chung Hsing University, Taichung, 40227 Taiwan; 3 Smart Sustainable New Agriculture Research Center (SMARTer), National Chung Hsing University, Taichung, 40227 Taiwan

Correction to: *Scientific Reports* 10.1038/s41598-022-17184-1, published online 27 July 2022

In the original version of this Article, Jason T. C. Tzen was omitted as a co-corresponding author. Correspondence and requests for materials should also be addressed to TCTZEN@dragon.nchu.edu.tw.

The original Article has been corrected.

