# Supplementary material for: Molecular characterization of polyphenol oxidase between small and large leaf tea cultivars
Source: Sci Rep. 2022 Jul 27;12:12870. doi: 10.1038/s41598-022-17184-1 (PMC9329367; doi:10.1038/s41598-022-17184-1)
Supplement: Supplementary file 1 — Supplementary Information. [file 41598_2022_17184_MOESM1_ESM.pdf]

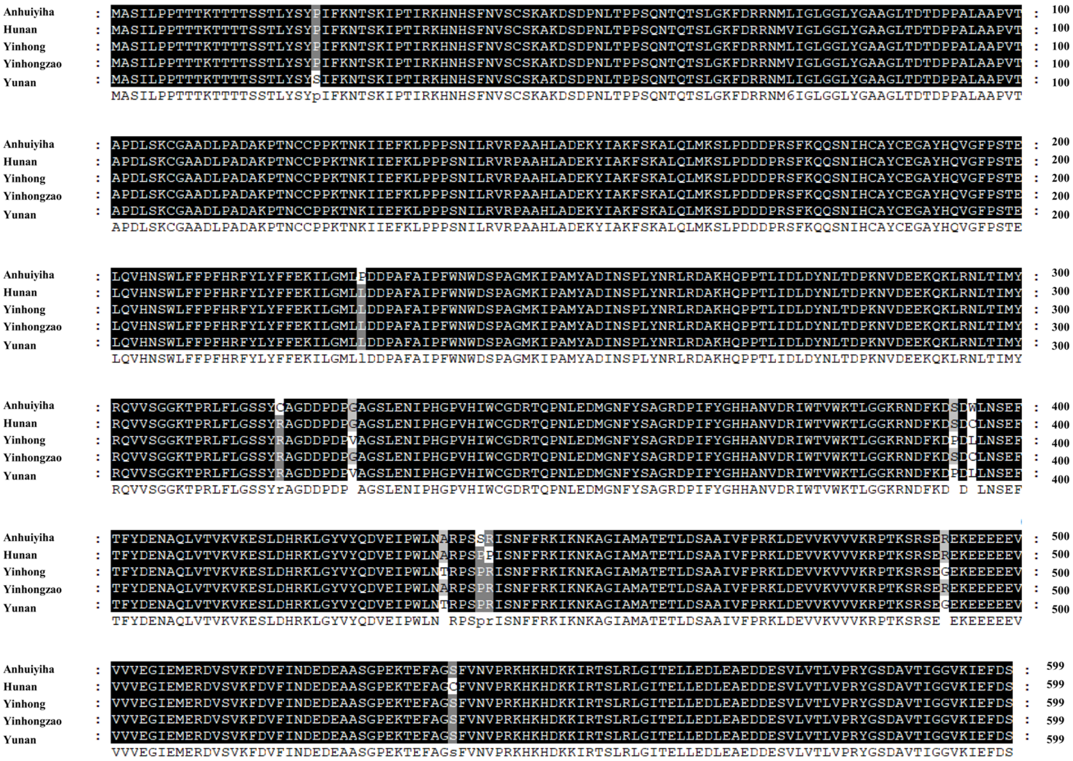

Fig. S1 Alignment of amino acid sequences of CsPPO in different tea cultivars. Black and gray shades indicate sequence conservations of 100% and >80%, respectively. Sequences were aligned using the ClustalW algorithm.

Table S1. List of primer sequences used for quantitative RT-PCR in this study

Primer name	Primer sequence (5'-3')
CsPPO1-Forward	5'-atggcttccattctccctccaacc-3'
CsPPO1-Reverse	5'-gatctcgtcccgctgagtagaag-3'
CsPPO2-Forward	5'-ataaccgtctccgtgacgc-3'
CsPPO2-Reverse	5'-tgcaggaaccgatcacaaca-3'
rCsPPO-Forward	5'-atctggacggtgtggaagac-3'
rCsPPO-Reverse	5'-aagctgagcattttcgtcgt-3'
rCsCHS-Forward	5'-ttgggtcagacccaattcc-3'
rCsCHS-Reverse	5'-aatgtaaggcccacttcacg-3'
rCsANS-Forward	5'-gagccgccaaaggagaagatt-3'
rCsANS -Reverse	5'-ccaagttcttgggatttccg-3'
rCsANR-Forward	5'-gctcaatctccatctcccacg-3'
rCsANR -Reverse	5'-tgaggaacttggctagctcgg-3'

ANS: anthocyanidin synthase. ANR, anthocyanidin reductase. CHS: chalcone synthase. PPO: polyphenol oxidase.
